# Ocular Response Analyzer

**DOI:** 10.5005/jp-journals-10008-1103

**Published:** 2012-10-16

**Authors:** Sushmita Kaushik, Surinder Singh Pandav

**Affiliations:** 1 Advanced Eye Centre, Postgraduate Institute of Medical Education and Research, Chandigarh, India; 2 Advanced Eye Centre, Postgraduate Institute of Medical Education and Research, Chandigarh, India

**Keywords:** Hysteresis, Ocular response analyser, Corneal biomechanics, Tonometry.

## Abstract

Until recently, corneal biomechanical properties could not be measured *in vivo.* The ocular response analyzer is a new, noninvasive device that analyses corneal biomechanical properties simply and rapidly. The ORA allows cornea compensated IOP measurements and can estimate corneal hysteresis (CH) and corneal resistance factor (CRF). It is designed to improve the accuracy of IOP measurement by using corneal biomechanical data to calculate a biomechanically adjusted estimate of intraocular pressure. This review critically evaluates the technology and its implications in current day glaucoma management.

## INTRODUCTION

Glaucoma is one of the leading causes of visual impairment and blindness worldwide.^1-3^ Lowering intraocular pressure (IOP) is the only proven means to slow or halt disease progression, as shown by studies of those at high risk of developing glaucoma [ocular hypertension treatment study (OHTS)],^4^ those with early to moderate glaucoma [collaborative initial glaucoma treatment study^5^ and early manifest glaucoma trial (EMGT)]^6^ and those with more advanced glaucoma [collaborative initial normal-tension glaucoma study^7^ and advanced glaucoma intervention study (AGIS)].^8^ Across all randomized, controlled trials, lowering IOP resulted in reduction in rates of worsening of glaucoma over 5 years.^8,9^ These studies confirm that one of the pathophysiological basis for glaucoma is elevated IOP.

Goldmann applanation tonometry (GAT) is regarded as the reference standard by which to measure IOP. GAT is known to be influenced by factors related to the corneal properties, such as corneal curvature and central corneal thickness (CCT).^10,11^ Although GAT may be less prone to biomechanical influence than Schiotz tonometry, it is clearly affected by corneal biomechanical influences, such as hydration, elasticity, hysteresis and rigidity. Achieving accurate estimates of intraocular pressure remains difficult. Even though increased IOP is the only proven means to delay or halt the development of glaucoma or progression of established disease, there remains the paradox of normal tension glaucoma with so-called ‘normal’ IOP and ocular hypertension with raised IOP but no disease. This has raised questions about factors other than IOP in the pathophysiology of glaucoma.

Until recently, corneal biomechanical properties could not be measured *in vivo.* The ocular response analyzer (ORA; Reichert Ophthalmic Instruments, Inc., Buffalo, NY, USA) is a new, noninvasive device that analyses corneal biomechanical properties simply and rapidly.^12,13^ The ORA allows cornea compensated IOP measurements and can estimate corneal hysteresis (CH) and corneal resistance factor (CRF). It is designed to improve the accuracy of IOP measurement by using corneal biomechanical data to calculate a biomechanically adjusted estimate of intraocular pressure. The ORA generates two separate IOP output parameters: Goldmann-correlated IOP (IOPg) and the corneal-compensated IOP (IOPcc).

## ORA

Reichert has produced an instrument, the ocular response analyzer, which measures the corneal response to indentation by a rapid air pulse. The principles of the ORA are based on those of noncontact tonometry, in which the IOP is determined by the air pressure required to applanate the central cornea. A fully automated alignment system positions an air tube to a precise position relative to the apex of the cornea. Once aligned, a 25 millisecond air pulse applies pressure to the cornea. The air pulse causes the cornea to move inward, past applanation and into a slight concavity before returning to normal curvature. Corneal deformation is recorded via an electro-optical infrared (IR) detection system (similar to the classical air-puff tonometers).

**Fig. 1:**
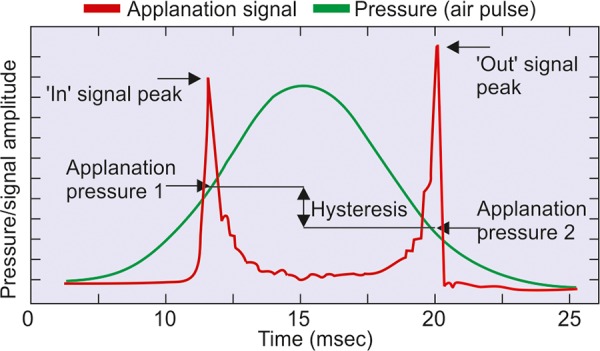
Measuring the biomechanical properties of the cornea

The ORA acquires corneal biomechanical data by quantifying this differential inward and outward corneal response to an air pulse over a time span of approximately 20 milliseconds. Once the air pulse induces the desired indentation/applanation, it symmetrically reverses, which allows the cornea to resume its original shape. Because, a time lag is necessary to activate the reversal of the air pulse, the cornea actually indents mildly beyond the intended applanation point. This action permits the detection of a second applanation point, as the cornea returns from its overapplanated state. Using the first applanation pressure point (P1) and the second applanation pressure point (P2), the ORA generates two separate IOP output parameters (Fig. 1).

### Goldmann-correlated IOP (IOPg)

This is the average of the inward (P1) and outward (P2) applanation pressures. This parameter is closely correlated with GAT-IOP.

### Corneal-compensated IOP (IOPcc)

Derived from both IOP and corneal biomechanical data.

The ORA supplies two additional parameters that reflect biomechanical properties of the cornea and demonstrate inter-individual variation.

### Corneal Hysteresis (CH)

During the ORA measurement process, the cornea absorbs some energy from the initial air pulse, which causes the second applanation pressure measurement to be lower than the initial measurement. The difference between the two pressures (P1-P2) is CH. This ORA parameter is thought to represent the viscoelastic nature of the cornea, or its ‘viscous-damping’ capacity.

### Corneal Resistance Factor (CRF)

The CRF is derived from the formula (P1-kP2) where k is the constant determined from an empirical analysis of the relationship between both P1 and P2 and CCT. CRF offers a measurement of corneal resistance.

The measurement signal consists of a green symmetric curve, which corresponds to the air-pulse pressure and a red asymmetric curve, which corresponds to applanation of the cornea via the signal produced by the IR detector. The red curve has two principal peaks, which correspond to points P1 and P2 on the green curve. P1 is the pressure at the first applanation event as the cornea moves inward under the increasing force of air pulse (inward applanation). P1 is similar to the air-pulse system usually used in noncontact tonometry to measure IOP. P2 is the pressure corresponding to the second applanation event as the cornea returns to its normal curvature under the decreasing force of the air pulse (outward applanation). Due to the dynamic nature of the measurement process, viscous damping in the cornea causes delays in the inward and outward applanation events (energy absorption). This results in two different pressure values at the inward and outward events, with the second outward applanation pressure always lower than the first inward applanation pressure. If abnormal corneal movements or surface irregularities exist, peaks may be lower, wider or otherwise irregular. If corneal structure is very abnormal, the whole red curve could be very irregular because of an abnormal mechanical response. Using this bidirectional applanation measurement, the ORA is able to present the four different parameters.

Viscous damping of the cornea may be important clinically, since increased damping capacity of the eye may effectively buffer potentially harmful IOP fluctuations. Theoretically, this improved buffering might result in reduced stress/strain on both the optic nerve and peripapillary scleral tissues. Woo et al^14^ have analyzed stress and strain characteristics of sections from different regions of whole human globes and found that the biomechanical characteristics of the anterior segment approximated that of whole globes. Wells et al^15^ reported significant correlation between laminar compliance and corneal hysteresis in glaucoma patients which runs counter to the association of lower corneal hysteresis associated with glaucoma and progression, as reported in other studies.^16^

Downs et al^17^ have described changes in the viscoelastic properties of the peripapillary sclera in normal monkey eyes and those with early glaucoma and have suggested that tissue viscoelastic properties change in the optic disk region of eyes exposed to chronic elevations in intraocular pressure. These findings may reflect an underlying predisposition to glaucoma change or a change that has occurred as a result of part of the glaucomatous process or they may reflect contributions from both. The damping effect of CH may be an explanation of why eyes with high CH measurements as in OHT are protected from developing glaucoma despite high IOP.

The relationship between IOP and corneal biomechanical properties is not understood well. A recent paper by Sun et al^18^ postulated that raised IOP itself could alter corneal biomechanics. They measured CH before and after IOP-lowering therapy including surgery, medications and laser in a cohort of chronic primary angle closure glaucoma patients and reported the CH recovering after IOP lowering. It is not clear from their paper whether any corneal edema secondary to the raised IOP pretreatment could have decreased the CH which reverted to normal when the IOP was controlled. Other authors have given alternative explanations to explain this effect including shallower indentation of the cornea by ORA at higher IOP levels^19,20^ owing to limitation of the air-jet of the ORA at higher pressures. They postulated that the CH measurement with the ORA alters with IOP at higher IOP levels.

The authors studied the corneal biomechanical properties across the spectrum of glaucoma.^21^ They found CH measurements were significantly less in primary open-angle glaucoma (POAG) and normal-tension glaucoma (NTG) compared to normal subjects (p = 0.034 and p = 0.030 respectively), regardless of the IOP. The CRF was significantly less in NTG and maximum in POAG and ocular hypertension (OHT). Regression analysis with CH as dependant variable showed significant association with GAT-IOP and CRF (p < 0.001) but not CCT, persisting on multivariate analysis (adjusted R squared = 0.483). GAT-IOP correlated strongly with Goldmann-correlated IOP on the ORA (IOPg) (r = 0.82; p < 0.001), but limits of agreement between the measurements were poor. CRF appeared to influence GAT-IOP measurements more than simple geometrical thickness measured by CCT.

Corneal biomechanical data appear to be a promising addendum to the complex issues of glaucoma occurrence and prognosis. Corneal factors, such as CCT, corneal hysteresis and CRF, may constitute a pressure-independent risk factor for glaucoma maybe related to the structure of the eyeball itself. If so, they may provide a clue to many unanswered questions, such as why some patients progress despite achieving ‘target IOP’, and also many cases of unexplained unilaterality of primary glaucoma. Further studies are warranted to validate these measurements. Questions remain on the reliability of ORA data in situations of high IOP and the effect of surgery, lasers and medications on corneal biomechanical measurements. However, IOP measurements from the ORA are not interchangeable with, and are unlikely to replace Goldmann applanation tonometry in the present time. We need to use these measurements judiciously in our day-to-day glaucoma practice.
